# MiR-489 inhibited the development of gastric cancer via regulating HDAC7 and PI3K/AKT pathway

**DOI:** 10.1186/s12957-020-01846-3

**Published:** 2020-04-13

**Authors:** Haiyan Zhang, Lingyun Li, Cuicui Yuan, Congcong Wang, Tiantian Gao, Zhiwei Zheng

**Affiliations:** 1 Department of Gastroenterology, People’s Hospital of Rizhao, Rizhao, 276800 China; 2 Department of Internal Medicine, Laishan Branch Hospital of Yantai Yuhuangding Hospital, Yantai, 264003 China; 3 Department of Cardiovascular Medicine, The People’s Hospital of zhangqiu area, Jinan, 250200 China; 4 Department of Operation Room, The People’s Hospital of zhangqiu area, Jinan, 250200 China; 5 Department of Nephrology, The People’s Hospital of zhangqiu area, Jinan, 250200 China; 6 Department of General Surgery, People’s Hospital of Rizhao, 126, Tai’an Road, Rizhao, 276800 China

**Keywords:** Gastric cancer, miR-489, HDAC7, PI3K/AKT pathway

## Abstract

**Background:**

Mounting evidences have displayed that the dysregulation of miRNAs plays important roles in the pathogenesis of gastric cancer (GC). The purpose of this study was to explore the biological functions and potential mechanism of miR-489 in GC progression.

**Methods:**

Quantitative real-time PCR (qRT-PCR) and western blot were performed to examine the mRNA expression and protein levels of miR-489 and HDAC7. The relationship between miR-489 and HDAC7 was analyzed by Spearman rank correlation. 3-(4,5-dimethylthiazolyl-2)-2,5-diphenyltetrazolium bromide (MTT) assay and transwell assays were conducted for determining the effect of miR-489 and HDAC7 on GC cell viability, migration, and invasion. TargetScan and luciferase reporter assay were used to confirm the target gene of miR-489 in GC cells.

**Results:**

The findings showed that miR-489 was dramatically decreased in GC tissues and GC cell lines (SGC-7901 and MKN45). Moreover, it was closely correlated with overall survival (OS) and progression-free survival (PFS) of GC patients. Downregulation of miR-489 significantly promoted GC cell proliferation, invasion, and migration. Additionally, HDAC7 was confirmed as the direct target of miR-489. Knockdown of HDAC7 exerted inhibited effect on GC progression and it markedly overturned miR-489 inhibitor-medicated effect on GC cells. More interestingly, via targeting HDAC7, miR-489 blocked the activation of PI3K/AKT pathway in GC cells.

**Conclusions:**

Correctively, miR-489 played as a tumor suppressor in GC cell growth by targeting HDAC7, and miR-489 might function as a novel biomarker for diagnosis or therapeutic targets of human GC.

## Background

Gastric cancer (GC) is the fourth of the most common malignant tumor in the world and the second leading cause of cancer-related human mortalities [1]. Presently, although the standard therapeutic treatments for GC including surgery, chemotherapy, and targeted therapy are effective, the long-term survival rate is still not ideal [2]. Due to the uncontrollable proliferation and metastasis of tumor cells, the prognosis of GC patients is very poor, so identification of novel and more efficient biomarkers for GC is extremely valuable [3].

MicroRNAs (miRNAs), 19 to 25 nucleotide non-coding RNA molecules, have shown can regulate the expression of various genes which involved in the carcinogenesis, metastasis, invasion, and recurrence of GC [4, 5]. MiRNAs are reported that they can modulate gene expression via binding to mRNAs 3′UTR, resulting in mRNA degradation or blocking mRNA translation. By targeting mRNA, multiple signaling pathways could be modulated by miRNAs, thereby participating in various cellular activities, including cell survival, differentiation, metastasis, and apoptosis [6, 7]. Growing evidences showed that miRNAs were involved in GC development as tumor suppressors or oncogenes. For example, miR-567 was downregulated in GC tissues and cell lines, miR-567 overexpression inhibited GC tumor growth through PIK3AP1-PI3K/AKT signaling pathway [8]. Wang J et al. found that miR-1224 upregulation suppressed GC metastasis via targeting FAK [9]. However, downregulation of miR-21 inhibited the growth and metastasis of GC cells, which may be a promotion effect on GC progression [10]. MiR-489 was proved to participate in the tumorigenesis of breast cancer, colorectal cancer, and glioma [11–13]. Moreover, Zhang B et al. displayed that miR-489 functions as a tumor suppressor in GC tumor growth and invasion [14]. Nevertheless, miR-489’ mechanism in the modulation of GC viability, migration, and invasion needs further investigation.

In our study, we found a lower expression of miR-489 in GC tissues and cells than that in the control groups. Moreover, upregulating miR-489 inhibited GC cell viability, invasion, and migration. In addition, HDAC7 was determined as the direct target of miR-489. Via targeting HDAC7, miR-489 overexpression suppressed PI3K/AKT pathway in GC cells. In summary, these findings provided the mechanism by which miR-489 regulated GC development.

## Materials and methods

### Clinical tissues

The fifty-two GC tissues along with matched surrounding normal gastric epithelial tissue samples applied in this study were obtained from People’s Hospital of Rizhao. Informed written consent was obtained from all of the patients. None of them have ever received any types of anti-cancer treatments. This study was approved by the Ethics Committee of People’s Hospital of Rizhao (no. 2014-07-0012). All GC tissues were stored in liquid nitrogen before further analysis.

### Cell lines and transfection

Two GC cell lines (SGC-7901 and MKN45) and the normal gastric epithelial cell line (GES-1) were maintained in RPMI-1640 medium containing with 10% FBS, and then cultured in an incubator with an atmosphere of 5% CO_2_ at 37 °C.

The miR-489 mimic/inhibitor, HDAC7 siRNA, and their corresponding negative controls were purchased from GeneCopoeia™ (Guangzhou, China). The transfection was conducted by using Lipofectamine 2000 Reagent (Invitrogen, USA).

### RT-PCR

Total RNA was isolated from GC tissues and cells via TRIzol reagent. The synthesis of cDNA was performed using M-MLV Reverse Transcriptase Kit. RT-PCR was performed using SYBR Green Real-Time PCR Assay Kit. U6 and GAPDH were served as internal control, and relative expression was calculated using 2-ΔΔCt method. The primer sequences used in this study were as follows: miR-489-F: 5′-ACACTCCAGCTGGGGTGACATCACATA-3′, miR-489-R: 5′-TGGTGTCGTGG AGTCG-3′; U6-F: 5′-ACACTCCAGCTGGGGTGCTCGCTTCGGCAGCACA-3′, U6-R: 5′-AGGGTCCGAGGTATTC-3′; HDAC7-F: 5′-AGGAGCAAGAACTTCGGC AA-3′, HDAC7-R: 5′-CACTGGGGTCCTGGTAGAAA-3′; GAPDH-F: 5′-AGTGTG ACGTTGACATCCGT-3′, GAPDH-R: 5′-GCAGCTCAGTAACAGTCCGC-3′.

### MTT assay

3-(4,5-dimethylthiazol-2-yl)-2,5-diphenyltetrazolium bromide (MTT) assay was performed to examine GC cell viability. GC cells and RPMI-1640 medium were seeded into 48-well plates at a density of 5 × 10^3^ cells/well and incubated for 24 h, 48 h, 72 h, and 96 h at 37 °C with 5% CO_2_. Then, MTT solution was added to incubate for another 4 h. Finally, enzyme-linked immunoassay was applied for measuring the absorbance at the OD 490 nm using the Multiskan Plate Reader (Thermo Fisher Scientific, Inc.).

### Transwell invasion and migration assay

The capacity of GC cells’ invasion and migration treated with miR-489 mimic, or inhibitor was detected with Transwell chambers (8 μm pore diameter, Corning, USA). Cells were put into the upper chamber of the plate at a density of 1 × 10^5^ cells with Matrigel (Franklin Lakes, NJ, USA). RPMI1640 mixed with 10% FBS was added into the lower chambers. After 24 h incubation, GC cells were passed through the polycarbonate membrane and fixed with 4% paraformaldehyde and stained with crystal violet, respectively. Under the CX-31 (Olympus, Tokyo, Japan), we counted from five fields in × 200 magnification. In vitro migration assay, it has no need for Matrigel.

### Western blot assay

We isolated the total protein samples from the cultured cells using RIPA lysis buffer supplemented with protease inhibitor (Beyotime). The concentration of the extracted protein was analyzed using BCA kit (Beyotime). The whole protein samples from cell lysates were separated by sodium dodecyl sulfate polyacrylamide gel electrophoresis system including 8–15% separation gel and 5% stacking gel, followed by electrotransferred to nitrocellulose membranes (PALL, New York, USA). After blocking with 5% skimmed milk powder for 2 h, the primary antibodies and secondary antibodies were added and incubated overnight at 4 °C and for 2 h, respectively. Finally, the enhanced chemiluminescence detection system was applied for the visual analysis. GAPDH was used as an internal control for the standardization of the proteins.

### Dual-luciferase assay

Bioinformatic analysis algorithm TargetScan (https://www.targetscan.org/vert_72/) was used to predict the targets of miR-489. Wild the binding region of HDAC7 was inserted into the pMIR-REPORT luciferase vector, named pMIR-HDAC7-wild. Mutated the binding site of HDAC7 in 3′UTR and cloned into the plasmid, named pMIR-HDAC7-mutant. MiR-489 mimic, miR-489 inhibitor, control mimic/control inhibitor, and luciferase plasmid pMIR-HDAC7 (wild-type/mutant, WT/MuT) were transfected into GC cells by using Lipofectamine 3000 (Invitrogen, CA). After cultivated 48 h, the luciferase activities were detected by Dual-Luciferase Reporter Assay System (Promega Corporation).

### Statistical analysis

The values were represented as the mean ± SD of three times in every experiment. Data were analyzed by the SPSS 22.0 statistical software and the statistics were performed by GraphPad Prism 6. Student’s *t* test was applied for comparing the difference between two groups. One-way analysis of variance (ANOVA) followed by Tukey’s test was carried out for multiple groups. Pearson’s correlation coefficient was applied for determining the correlation between miR-489 and HDAC7. Log-rank test was applied for analyzing the survival rate. A *P* < 0.05 was considered as statistically significant.

## Results

### MiR-489 was downregulated in GC tissues and cell lines

**Fig. 1:**
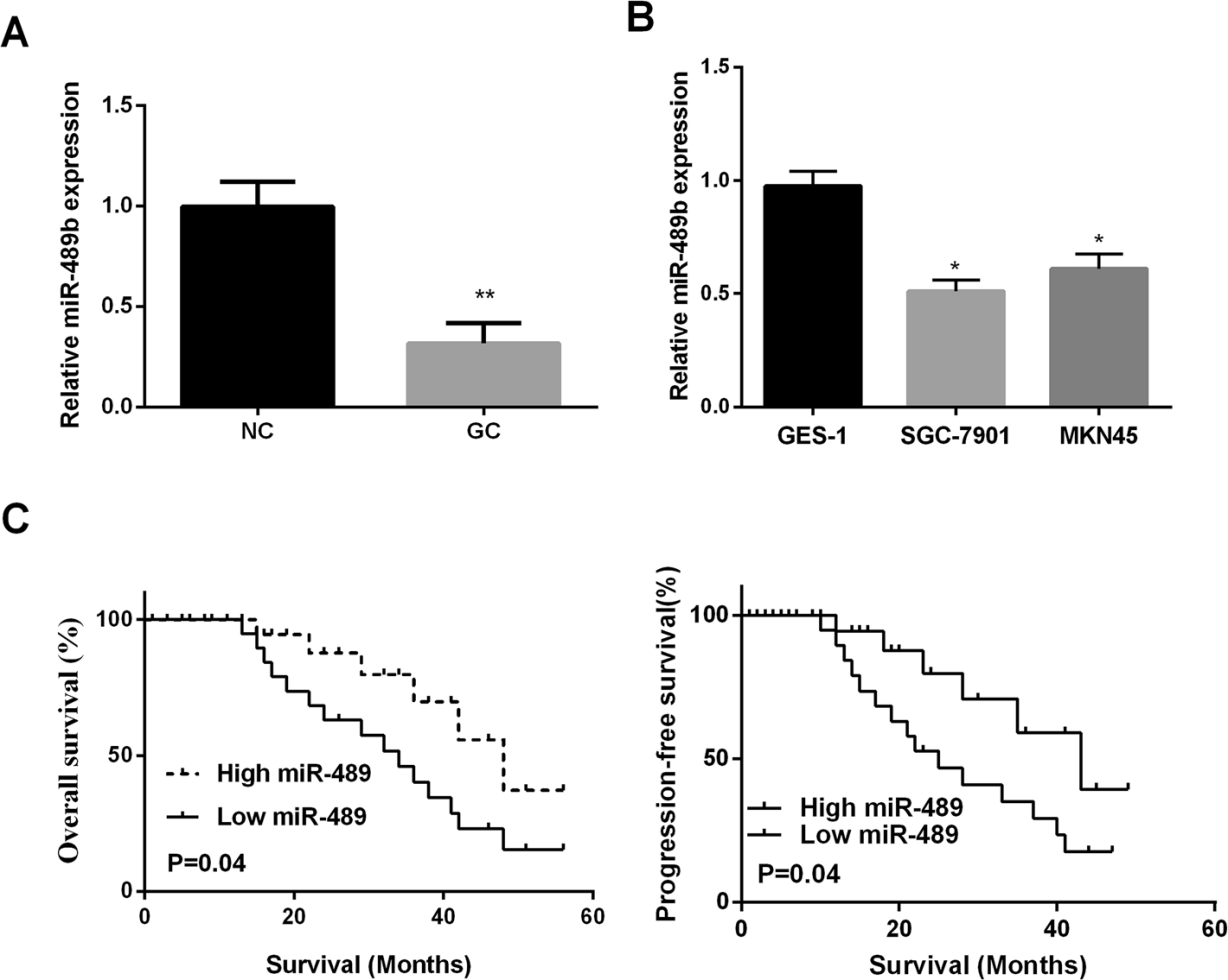
Reduce expression of miR-489 in GC is associated with poor prognosis. **a** Decreased of miR-489 in GC tissues (*n* = 52) was measured by qRT-PCR. **b** miR-489 expression was assessed by qRT-PCR in gastric cancer cell lines SGC7901, MKN45, and immortal gastric epithelial cells (GES-1). **c** Kaplan-Meier curve for overall survival and progression-free survival of GC patients with high or low expression of miR-489. *P < 0.05, **P < 0.01

RT-PCR was applied for measuring miR-489 expression in 52 paired GC tissues and cell lines. The findings displayed that miR-489 expression in GC tissues was observably lower than normal tissues (Fig. 1a). Additionally, our results show that miR-489 level is reduced in all two GC cell lines compared with that in GES-1 cells (Fig. 1b). Next, miR-489's clinical significance was investigated in GC patients. High or low expression of miR-489 in GC patients was divided by the median expression of miR-489. We found that miR-489 was closely associated with tumor size and differentiation (Table 1). Especially, results of Kaplan-Meier analysis revealed that low expression of miR-489 exhibited a poorer overall survival and progression-free survival of GC patients than these with high expression of miR-489 (Fig.1c). These data indicated that miR-489 might be an indicator for the prognosis of GC patients.

**Table 1:** Clinicopathological variables and the expression of miR-489 in gastric cancer patients

Item	Cases	miR-489		*P* value
		High	Low	
**Age (years)**				0.790
≥ 60	20	8	12	
< 60	32	14	18	
**Gender**				0.575
Male	33	13	20	
Female	19	9	10	
**TNM stage**				0.0008**
I–II	17	6	11	
III–IV	35	16	19	
**Tumor size**				0.049*
< 4 cm	16	10	6	
≥ 4 cm	36	12	24	
**Lymph node metastasis**				0.746
Yes	25	10	15	
No	27	12	15	
**Distant metastasis**				0.947
Yes	21	9	12	
No	31	13	18	
**Differentiation**				0.024*
Low	37	12	25	
Well	15	10	5	

MiR-489 overexpression suppressed GC cell viability, invasion, and migration.

**Fig. 2:**
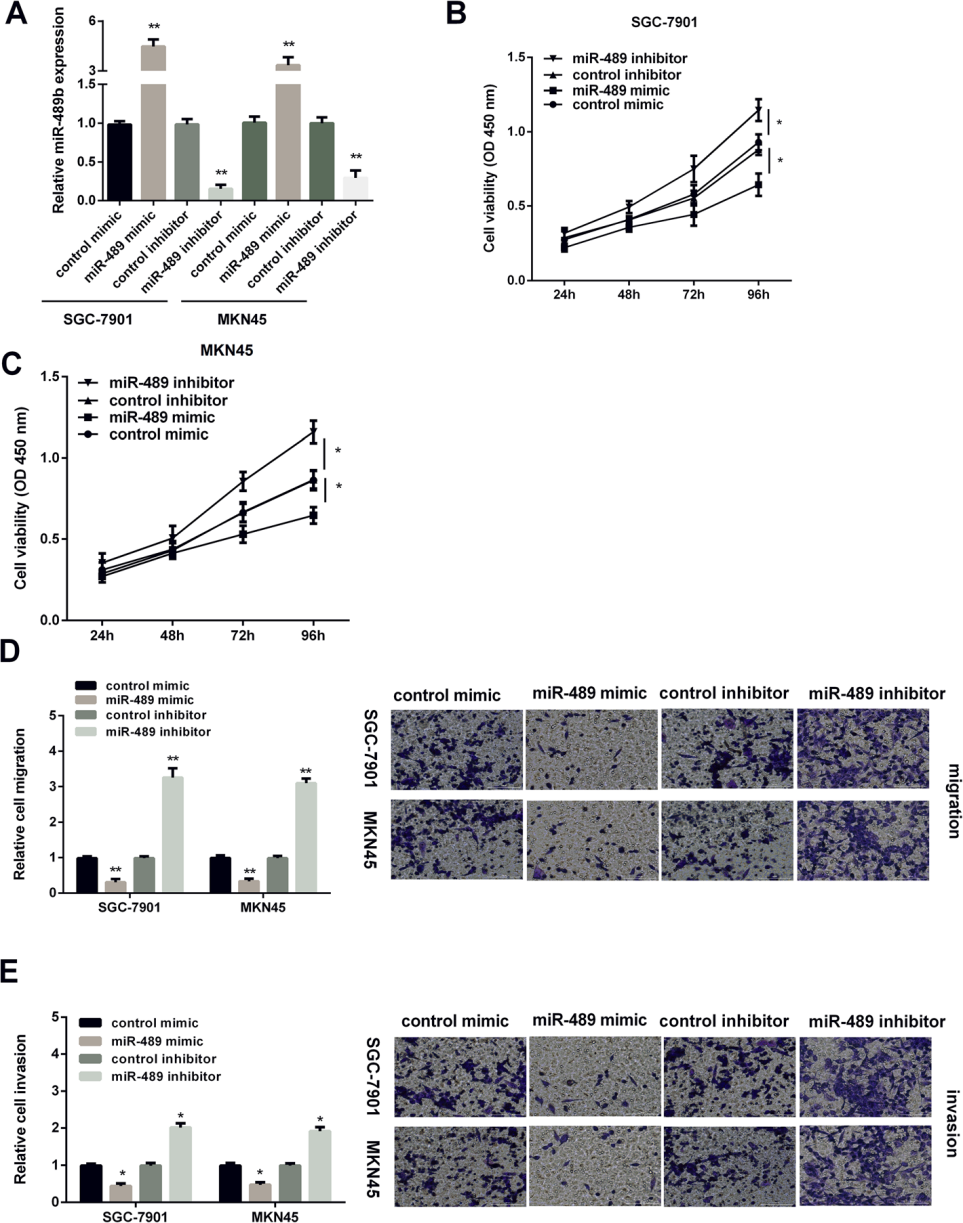
MiR-489 inhibited GC cell viability, invasion, and migration. **a** Expression of miR-489 in SGC7901 and MKN45 cell lines was measured by qRT-PCR post miR-489 mimic, miR-489 inhibitor, and control transfection. **b**, **c** CCK-8 assay of proliferation in SGC7901 and MKN45 cells following a time course of transfection with miR-489 mimic, miR-489 inhibitor, and control transfection. **d** Cell migration and **e** cell invasion in GC cells after transfection with miR-489 mimic, miR-489 inhibitor, and control transfection. *P < 0.05, **P < 0.01

RT-PCR was applied for measuring miR-489 expression in GC cells after treated with miR-489 mimic or inhibitor. As Fig. 2 a presented, miR-489 expression was obviously increased by miR-489 mimic, while decreased by miR-489 inhibitor in GC cells. MTT assay was applied for determining the effect of miR-489 on GC cell viability. The findings revealed that the cell viability was suppressed by miR-489 overexpression, but promoted by miR-489 silencing in GC cells (Fig. 2b, c). Transwell assays were applied for detecting miR-489 effect on GC cell invasion and migration. Results displayed that overexpression of miR-489 inhibited the migration of GC cells, whereas silencing of miR-489 enhanced cell migration (Fig. 2d). Moreover, miR-489 presented the similar effect on GC cell invasion (Fig. 2e). The above findings suggested that miR-489 overexpression showed inhibitory effect on GC development.

### HDAC7 was the direct target of miR-489

**Fig. 3:**
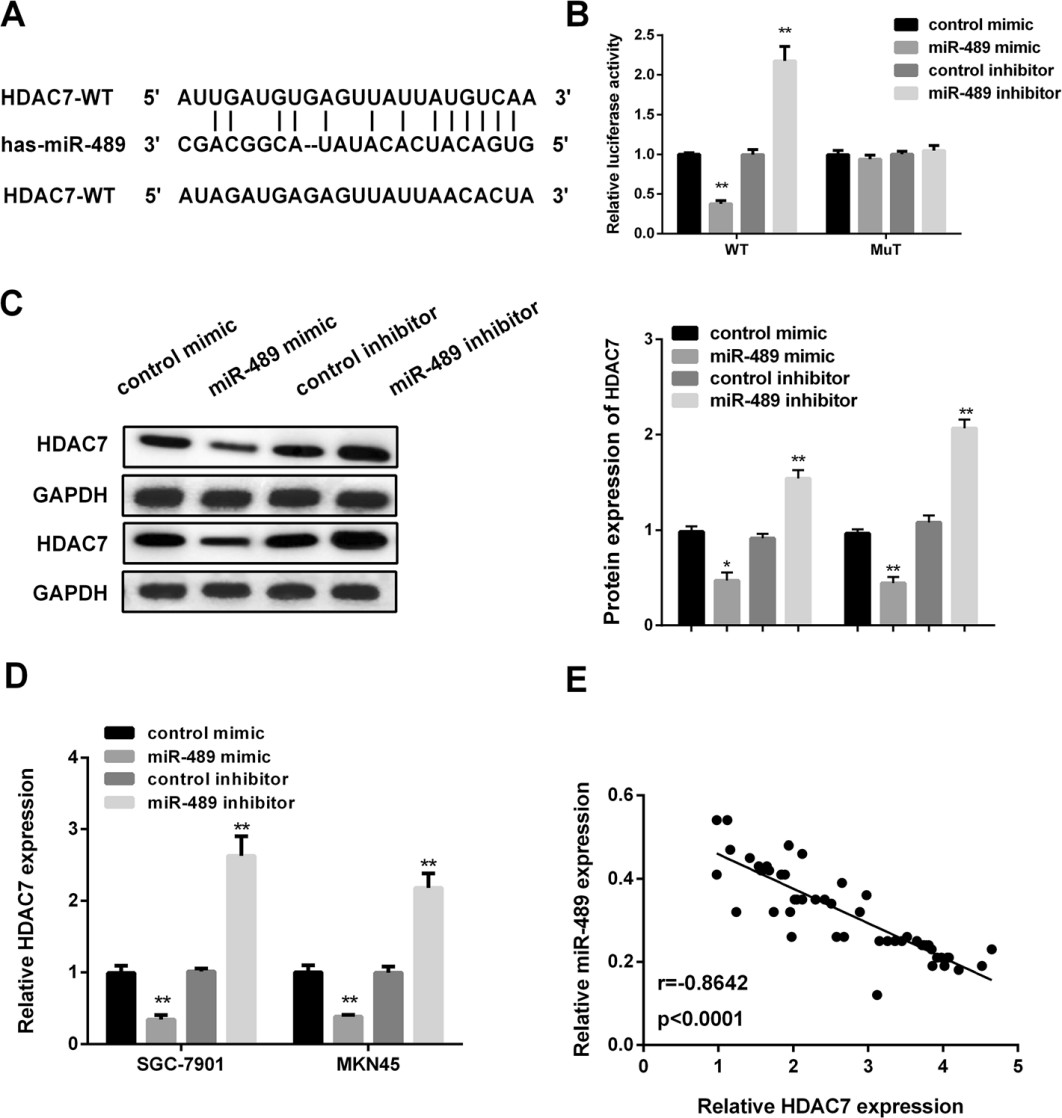
HDAC7 was the target of miR-489. **a** The schematic of the putative targeting sites in the HDAC 3′UTR with miR-489. **b** Luciferase activity of HDAC7 3′UTR-WT or -MuT in GC cells treated with miR-489 mimic, miR-489 inhibitor, and control. **c** HDAC7 protein level. **d** HDAC7 mRNA level in GC cells treated with miR-489 mimic miR-489 inhibitor, and control. Western blots have been performed three times. **e** miR-489 was inversely associated with HDAC7 expression. (*r* = − 0.8642, *P* < 0.001). *P < 0.05, **P < 0.01

The microRNA prediction website http://www.targetscan.org (TargetScan) was performed to predict the possible targets of miR-489. As we saw in Fig. 3 a, the results showed that the 3′UTR region of HDAC7 provided a binding site for miR-489. Subsequently, luciferase reporter assay was applied for further confirming the regulatory relationship between miR-489 and HDAC7. As Fig.3 b displayed, miR-489 mimic significantly reduced, while miR-489 inhibitor obviously raised the luciferase activity of HDAC7-3′UTR-WT. However, when combined with HDAC7-3′UTR-MuT reporter, there was no significant change in luciferase activity. Next, RT-PCR and western blot was used for examining HDAC7 level affected by miR-489. As Fig. 3 c and d presented, HDAC7 mRNA and protein level were decreased by miR-489 overexpression, while elevated by miR-489 silencing in GC cells. More importantly, miR-489 expression was negatively related to HDAC7 expression detecting by Pearson’s correlation coefficient (Fig. 3e). The value indicated that HDAC7 was the direct target of miR-489 in GC.

### HDAC7 was over-expressed in GC tissues

**Fig. 4:**
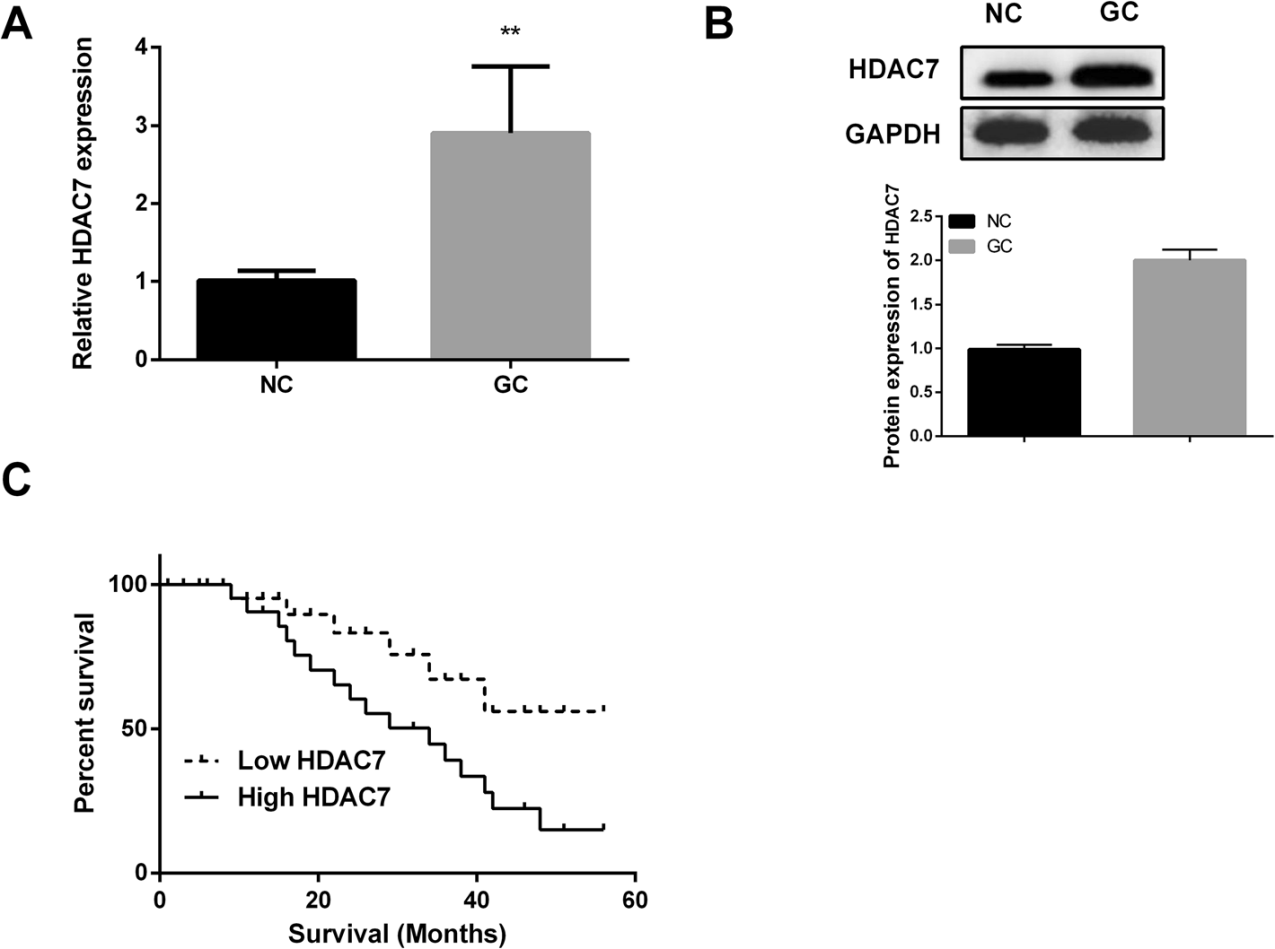
HDAC7 was closely related to the prognosis of GC patients. **a** The relative mRNA expression of HDAC7 in GC tissues (*n* = 52) was measured by qRT-PCR. **b** The relative protein expression of HDAC7 in GC tissues was determined by western blotting. Western blots have been performed three times. **c** Kaplan-Meier curve for 5-year survival time in patients with GC. *P < 0.05, **P < 0.01

RT-PCR and western blotting were applied for measuring HDAC7 expression in 52 pairs of GC tissues. Results displayed that HDAC7 mRNA level was observably higher in GC tissues compared to that in normal tissues (Fig. 4a). The similar results were observed in Fig. 4 b. Next, GC patients with high or low expression of HDAC7 were divided by the median expression of HDAC7. Kaplan-Meier analysis revealed that GC patients with high HDAC7 expression showed a poorer survival time than that with low HDAC7 expression (Fig. 4c). The findings demonstrated that HDAC7 might play critical role in GC development.

### HDAC7 reversed miR-489 effect on GC development

**Fig. 5:**
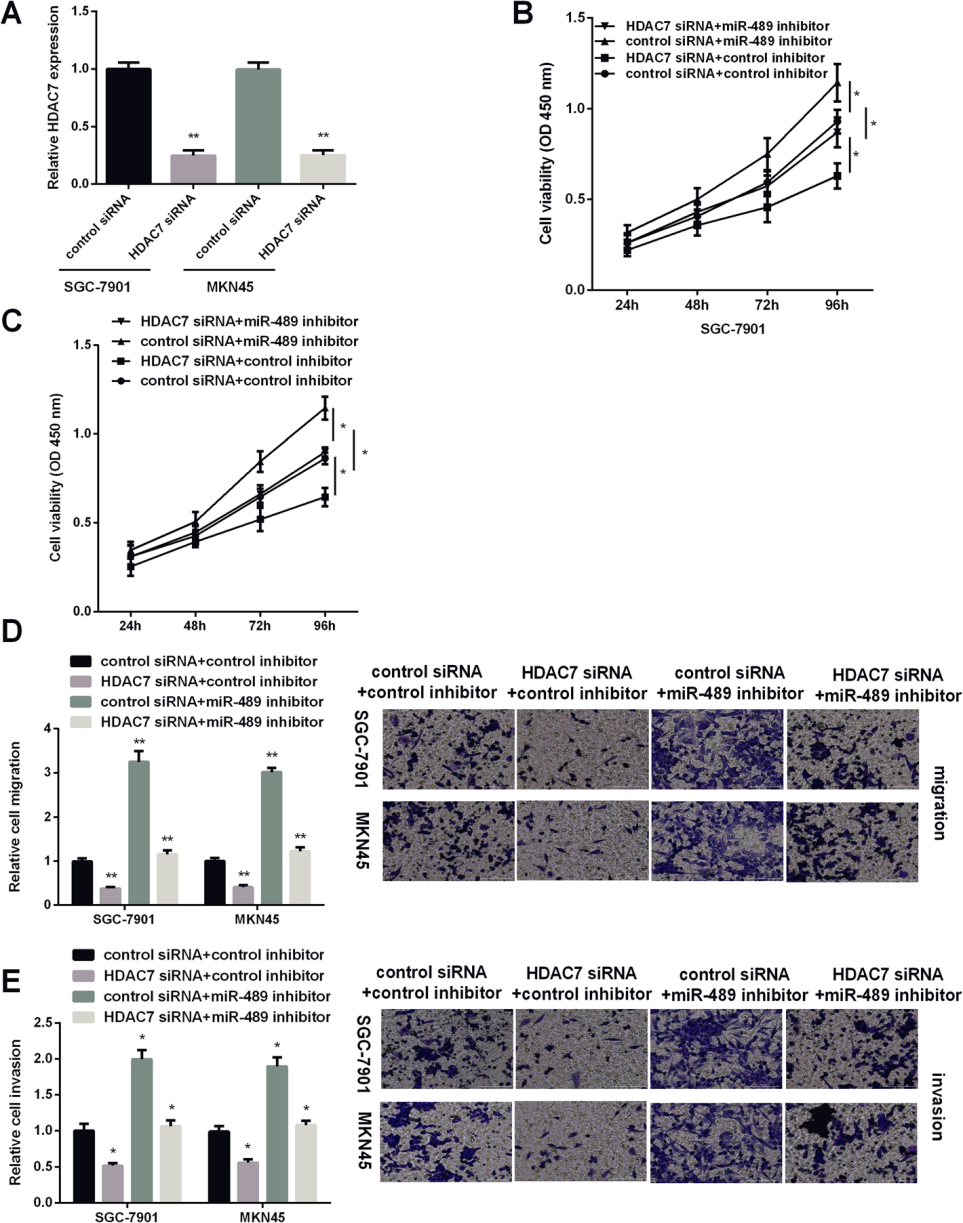
Effects of HDAC7 silencing on cell viability, invasion, and migration. **a** The relative mRNA expression of HDAC7 in SGC7901 and MKN45 cells was determined by RT-PCR after transfection of control siRNA (NC) or a specific HDAC7 siRNA (HDAC7 siRNA) in SGC7901 and MKN45 cells. **b**, **c** Cell viability, **d** cell migration, and **e** cell invasion in GC cells treated with HDAC7 siRNA, miR-489 inhibitor, or combined with HDAC7 siRNA. *P < 0.05, **P < 0.01

GC cells were treated with HDAC7 siRNA, miR-489 inhibitor, or miR-489 inhibitor combined with HDAC7 siRNA to investigate HDAC7 effect on miR-489 in the regulation of GC development. As Fig. 5 a shown, HDAC7 level was decreased when the GC cells were treated with HDAC7 siRNA. MTT assay results showed that downregulation of HDAC7 decreased GC cell viability, while miR-489 downregulation increased cell viability. Moreover, HDAC7 siRNA could attenuate miR-489 inhibitor effect on GC cell proliferation (Fig. 5b, c). Transwell migration analysis results showed that decreasing HDAC7 suppressed GC cell migration, and knockdown of miR-489 and HDAC7 in GC cells showed an obviously decreased cell migration compared with knockdown of miR-489 alone (Fig. 5d). The findings of transwell invasion assay were similar to cell migration experiments. MiR-489 inhibitor exerted promoted effect, while HDAC7 siRNA showed suppressed effect on GC cell invasion. Moreover, HDAC7 could attenuate miR-489 effect on cell invasion (Fig. 5e). Collectively, HDAC7 displayed an opposite effect of miR-489 on GC development.

### MiR-489/HDAC7 axis regulated EMT and PI3K/AKT pathway in GC cells

**Fig. 6:**
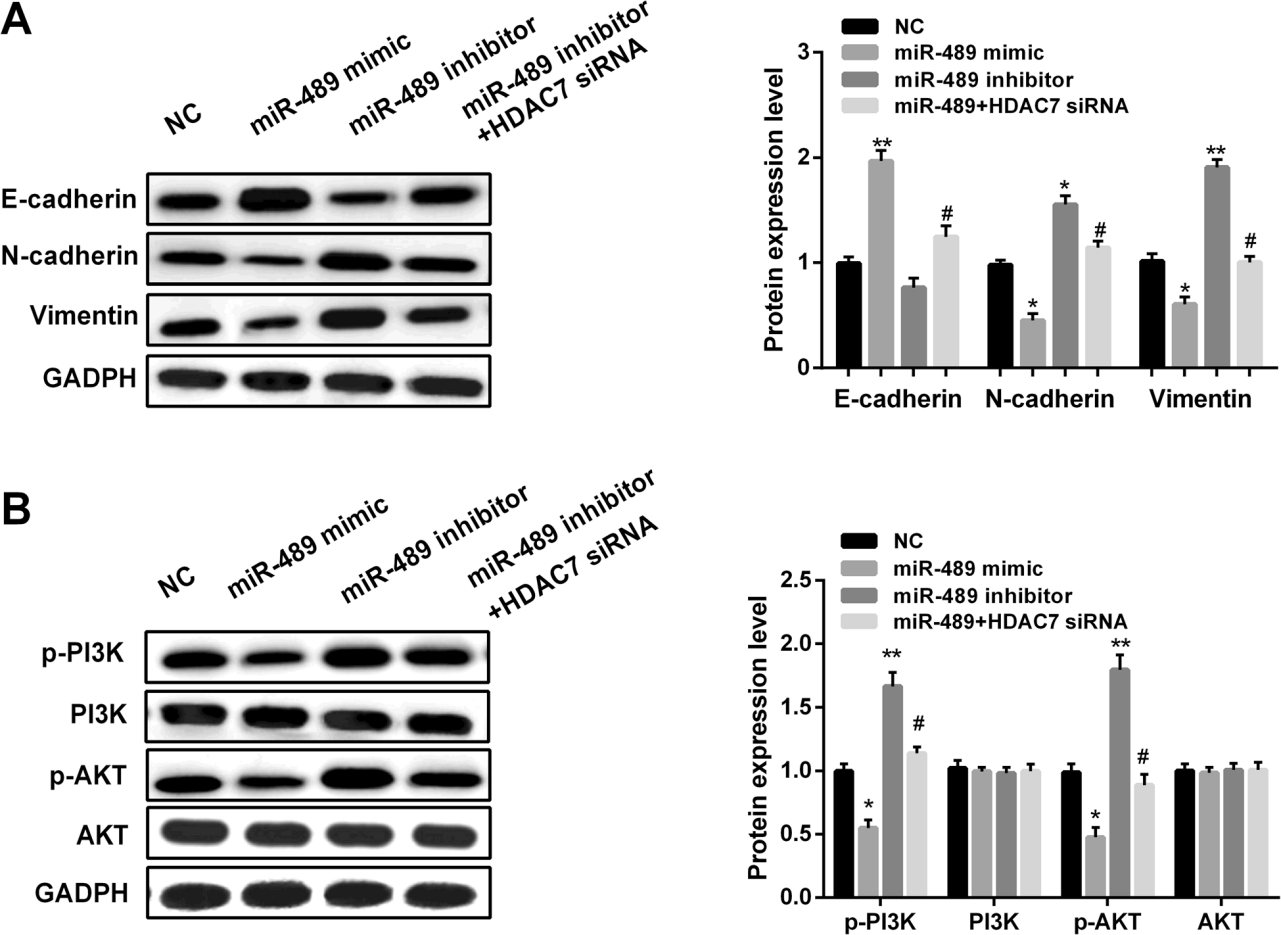
miR-489 modulated EMT and PI3K/AKT pathway. **a** E-cadherin, N-cadherin, and Vimentin protein levels in GC cells treated with miR-489 mimic, inhibitor, or combined with HDAC7 siRNA. *P < 0.05, **P < 0.01 vs. NC group; #*P* < 0.05 vs. miR-489 mimic group. **b** p-PI3K, PI3K, and p-AKT, AKT protein levels in GC cells treated with miR-489 mimic, inhibitor, or combined with HDAC7 siRNA. Western blots have been performed three times. *P < 0.05, **P < 0.01 vs. NC group; #*P* < 0.05 vs. miR-489 mimic group

HDAC7 was found to activate PI3K/AKT pathway in multiple tumors’ development. Here, we investigated the effect of miR-489 on EMT and PI3K/AKT pathway in GC cells. The levels of EMT-related markers and the downstream genes of PI3K/AKT pathway were measured by western blot. As we saw in Fig. 6 a, overexpression of miR-489 facilitated the level of E-cadherin, while inhibited N-cadherin and Vimentin levels in GC cells. However, downregulation of miR-489 showed the opposite effect on EMT-related markers. Moreover, knockdown of HDAC7 expression overturned the effect of miR-489 inhibitor on EMT. Additionally, miR-489 upregulation inhibited, miR-489 downregulation facilitated, and combined with HDAC7 downregulation attenuated the downstream genes of PI3K/AKT pathway, including p-PI3K, PI3K, and p-AKT, AKT (Fig. 6b). These data indicated that miR-489 inhibited GC development through PI3K/AKT pathway by targeting HDAC7.

## Discussion

Gastric cancer is a serious threat to human health. Currently, the incidence of gastric cancer is the fourth highest in the world, and it has the second highest mortality rate [15]. First choice in the treatment of gastric cancer is surgery [16], but because of early gastric cancer clinical manifestation is not obvious, medical screening mechanism is imperfect, diagnosis of gastric cancer rate is low in our country, terminal patients are likely to lose operation time or miss the optimal timing of surgery [17]. Accumulating evidences have displayed that the dysregulation of miRNAs served key roles in tumors’ development [18–20]. In this study, results showed a decreased miR-489 in GC tissues and cells and miR-489 served as an indicator for the prognosis of GC patients. Moreover, miR-489 inhibitor displayed facilitating effect on GC development. Additionally, HDAC7 acted as a direct target of miR-489. More importantly, PI3K/AKT pathway was modulated by miR-489/HDAC7 axis in GC cells.

MiR-489 was proved to take part in a variety of tumors’ development as a tumor suppressor. For instance, miR-489 re-expression suppressed the tumorigenesis of breast cancer [21]. Moreover, Gao S et al. displayed that miR-489 repressed tumor growth and the invasion of colorectal cancer [12]. Also, miR-489 showed the suppression effect on glioma cell proliferation and facilitating effect on cell apoptosis [13]. Here, in this study, we displayed that miR-489 showed an inhibitory effect on GC development as well. Restoration of miR-489 repressed GC cell viability, invasion, and migration, while knockdown of miR-489 facilitated GC progression.

Currently, the molecular mechanism of miR-489 in the regulation of GC development is not fully understood. TargetScan database was applied for predicting miR-489’s target gene and the results showed that HDAC7 was one of the candidate target genes of miR-489. Histone deacetylase HDAC7 is proved to be involved in a variety of cellular physiological processes and as a novel target for tumor therapy [22–24]. Previous studies have showed that HDAC7 was involved in the malignant phenotype of glioma regulated by ZNF326 [25]. Sang Y et al. displayed that HDAC7 facilitated cell growth and metastasis of lung cancer [26]. Moreover, Peixoto P et al. found that inhibiting HDAC7 reduced the tumorigenic activity of human glioblastoma and provided a new opportunity for tumor treatment [27]. Yu Y et al. have shown that high HDAC7 expression in cancerous gastric tissues correlates with distant metastasis and predicts a poor prognosis for patients with gastric cancer [28]. However, the regulatory effect of HDAC on GC has not been elucidated to a large extent. In this study, we revealed that HDAC was increased obviously in GC tissues and cells and displayed the opposite effect of miR-489 on GC development. More importantly, knockdown of HDAC7 overturned miR-489 inhibitor effect on facilitating GC cell viability, invasion, and migration.

The activation of PI3K/AKT pathway has been reported to take part in tumor cell proliferation and apoptosis [29–31]. Here, we proved that knockdown of miR-489 promoted the activation of PI3K/AKT signaling pathway and miR-489 restoration showed the opposite effect.

## Conclusion

All in all, miR-489 expression was decreased in GC while HDAC7 was increased and overexpression of miR-489 suppressed GC cell viability, invasion, and migration. HDAC7 was a target of miR-489 in regulating GC progression and miR-489 achieved the suppressed effect on GC by regulating PI3K/AKT signaling pathway.

## Data Availability

The datasets used and/or analyzed during the present study are available from the corresponding author on reasonable request.
